# Emerging Vector-Borne Zoonoses: Eco-Epidemiology and Public Health Implications in India

**DOI:** 10.3389/fpubh.2014.00168

**Published:** 2014-09-30

**Authors:** Ramesh C. Dhiman

**Affiliations:** ^1^National Institute of Malaria Research, Indian Council of Medical Research, New Delhi, India

**Keywords:** zoonotic diseases, scrub typhus, cutaneous leishmaniasis, Public Health, India, chikungunya virus, *P. knowlesi*, vector-borne disease

## Abstract

The diseases originating from animals or associated with man and animals are remerging and have resulted in considerable morbidity and mortality. The present review highlights the re-emergence of emerging mainly zoonotic diseases like chikungunya, scrub typhus, and extension of spatial distribution of cutaneous leishmaniasis from western Rajasthan to Himachal Pradesh, Kerala, and Haryana states; West Nile virus to Assam, and non-endemic areas of Japanese encephalitis (JE) like Maharashtra and JE to Delhi; Crimean–Congo hemorrhagic fever making inroads in Ahmedabad; and reporting fifth parasite of human malaria with possibility of zoonosis have been highlighted, which necessitates further studies for prevention and control. Emphasis has been given on understanding the ecology of reservoir hosts of pathogen, micro niche of vector species, climatic, socioeconomic risk factors, etc. Development of facilities for diagnosis of virus from insects, reservoirs, and human beings (like BSL4, which has been established in NIV, Pune), awareness about symptoms of new emerging viral and other zoonotic diseases, differential diagnosis, risk factors (climatic, ecological, and socioeconomic) and mapping of disease-specific vulnerable areas, and mathematical modeling for projecting epidemiological scenario is needed for preparedness of public health institutes. It is high time to understand the ecological link of zoonotic or anthroponotic diseases for updated risk maps and epidemiological knowledge for effective preventive and control measures. The public health stakeholders in India as well as in Southeast Asia should emphasize on understanding the eco-epidemiology of the discussed zoonotic diseases for taking preventive actions.

## Introduction

Most of the diseases of human beings, which are transmitted from man to man through an arthropod/insect or other invertebrate or vertebrates, are the diseases originated from animals. The socioeconomic development leading to clearing of forests, reclaiming wasteland for urbanization, etc. are resulting in ecological change leading to altered epidemiology of diseases. The changes in habitats of wild animals lead to man–vector contact, thus posing a risk to public health. In last two to three decades, many diseases, which were either forgotten or were restricted to a few foci, have re-emerged with vast spatial distribution. In recent years, there is increased awareness about resurgence of zoonotic or other re-emerging diseases in India (1). In the present review, the major vector-borne, zoonotic diseases of public health importance, which have re-emerged in India in last two to three decades, are being dealt with from the viewpoint of eco-epidemiology and public health implications. Of six major vector-borne diseases in India, i.e., malaria, dengue, chikungunya, filariasis, Japanese encephalitis (JE), and leishmaniasis, JE and cutaneous leishmaniasis (CL) are zoonotic. Malaria that was considered basically an anthroponotic disease has recently been investigated as zoonotic disease with evidence of *Plasmodium knowlesi* parasite from human cases from Southeast Asia. Scrub typhus and leptospirosis are spreading to new areas. Crimean–Congo hemorrhagic fever (CCHF) has been reported from India for the first time in 2010 while West Nile virus (WNV) is making inroads into new areas. Swine flu, which was not known till 2009, emerged in pandemic form throughout the world. The details of epidemiological aspects of malaria, CL, and scrub typhus as parasitic diseases; and JE, WNV, chikungunya, and CCHF as arboviral diseases in the context of changing ecology and transmission pattern are discussed in the present review so as to highlight the importance of eco-epidemiology, public health implications, and way forward for preparedness to combat their further spread. The general information in respect of seven vector-borne zoonotic diseases, which have re-emerged in last two to three decades are summarized in Table 1.

**Table 1:** **Major emerging/re-emerging zoonotic diseases in India**.

	Disease	First reporting in India	Geographic distribution	Pathogen	Vector species	Animals involved	Preferred ecological conditions
1	Malaria (zoonotic)	2013 [Tyagi et al. (2)]	Andaman and Nicobar Islands	*Plasmodium knowlesi*	Not known	Monkeys in Malaysia	Forest ecosystem
2	Cutaneous Leishmaniasis	1971 [WHO (3)]	Rajasthan, Himachal Pradesh, Kerala, Haryana	*Leishmania tropica, L. donovani* (in Himachal Pradesh)	*Phlebotomus salehi* and *P. papatasi/P. sergenti*	Rodent, dog	Zoonotic cycle in agricultural field with rodent burrows
3	Scrub typhus	1934 [From Vivekanandan et al. (4)]	Whole country is reportedly endemic	*Orientia (Rickettsia) tsutsugamushi*	*Leptotormbidium deliense*	*Rattus rattus*, mice	Moist, scrubby vegetation, coinciding with distribution of *Rattus rattus*
4	Japanese Encephalitis	1955 (http://www.icmr.nic.in/pinstitute/niv/JAPANESE%20ENCEPHALITIS.pdf)	Rice growing areas, Uttar Pradesh, Karnataka, West Bengal, Assam, Bihar etc.	JE virus	Culex spp. of mosquitoes, Anopheles	Pigs, egret birds	Rice fields
			Report from Delhi in 2011.	
5	West Nile	1952 [Paramasivan et al. (5)]	Mainly southern India, recent reports from Assam, Maharashtra	WN virus	*Culex vishnui* group of mosquitoes	Birds	River fields
6	Chikungunya	1824 (http://www.searo.who.int/entity/emerging_diseases/topics/Chikungunya/en)	Mainly southern India	CHIK virus	*Aedes aegypti, A. albopictus*	Not known in India (monkeys in Africa, Malaysia)	Not fully understood
7	Crimean–Congo hemorrhagic fever	2010 [Patel et al. (6), Yadav et al. (7)]	Gujarat	CCHF virus	*Hyalomma anatolicum anatolicum*	Livestock	Rural environment in vicinity of livestock

## Parasitic Zoonotic Diseases

### Malaria

Malaria is a parasitic disease caused by four species of Plasmodium parasite, i.e., *P. vivax, P. falciparum, P. malariae*, and *P. ovale* and transmitted by anopheline mosquitoes. In addition, rodent malaria parasites (*P. berghei*, and *P. chabaudi*) and avian malaria parasite (*P. gallinaecium*) are also circulating in nature.

Singh et al. (8) in 2004 for the first time reported a naturally acquired focus of *P. knowlesi* in human beings in Malaysia. Further, the group (9) described the first focus of human malaria infections with *P. knowlesi* and demonstrated from Kapit Division of Sarawak, Malaysian Borneo, that wild monkeys in the forest were infected with malaria parasite including *P. knowlesi*. The number of *P. knowlesi* genotypes per infection was much higher in monkeys than human beings, providing circumstantial evidence that *P. knowlesi* transmitted malaria is essentially a zoonotic disease. In such a scenario, deforestation and intrusion of man into forests may result into devastating outbreaks of malaria in Malaysia. Recently Tyagi et al. (2) while studying drug resistant-associated marker genes in *P. falciparum* in Andaman and Nicobar Islands (India), found co-infections with *P. knowlesi* up to the tune of 11.9% of which 5.3% were mono-infections with *P. knowlesi* only. The study emphasized that in South Asia, larger population might be at risk of *P. knowlesi* infection and such co-infections with *P. falciparum* would warrant reconsideration of malaria drug policy. However, it is assumed that in India also *P. knowlesi* infection is a zoonotic one. In Andaman and Nicobar Islands, the human dwellings are close to forest area, thus, necessitating the need for understanding role of monkeys in the epidemiology of *P. knowlesi* transmitted malaria in India for advocating preventive and control measures.

Spence et al. (10) demonstrated efficient and reproducible vector transmission of *P. c. chabaudi*, a rodent malaria parasite, through *Anopheles stephensi*. In view of changing ecological scenario, the malaria parasites of animals might be adapted to human akin to *P. knowlesi*.

### Leishmaniasis

In India, there are two forms of leishmaniasis, visceral and cutaneous. Visceral leishmaniasis (VL), commonly known as Kala-azar, is basically an anthroponotic disease and is confined mainly to Bihar, Jharkhand, West Bengal, and Uttar Pradesh. The vector species is *Phlebotomus argentipes*. However, the occurrence of cases of VL from Himachal Pradesh (11), Uttarakhand (12), resurgence from Assam since 2004^1^, and detection of *L donovani* from cases of CL from Himachal Pradesh (13) highlights the need for understanding the epidemiology of VL, which could be due to zoonotic reservoir in such erstwhile non-endemic areas of leishmaniasis.

In India, the known focus of CL is western Rajasthan (3), wherein the reservoir of the infection is *Meriones hurrianae*, a desert rodent in rural areas, and the vector is *P. salehi*. On the other hand, in urban area, dogs are reservoir and *P. papatasi/P. sergenti* are the vectors. Man contracts CL accidentally while working/staying in agriculture fields. Owing to lack of knowledge about the signs and symptoms of CL, the incidence is not known resulting into neglect of the problem. The detection of a new focus of CL from Himachal Pradesh postulated the role of zoonotic reservoir, increased man to vector contact due to deforestation and construction activities in the area (14). Recording of stray cases of CL from Kerala, Assam, and the state of Haryana in India highlights the need to study the role of zoonotic reservoir in VL and CL transmission in Himachal Pradesh and epidemiological investigation of other foci from the viewpoint of ecological conditions for possible reservoir and sand fly vectors.

### Scrub typhus

Scrub typhus is caused by a rickketsia, *Orientia tsutsugamushi* and transmitted by a trombiculid mite, *Leptotrombidium deliense*. As the name suggests, the disease is confined to scrubby/jungle areas and typhus word means typos (greek word), i.e., fever. The word tsutsugamushi is a Japanese word; tsutsuga means dangerous and mushi means insect/mite. The geographic distribution is confined to Southeast Asia including Japan and Korea. In India, the disease was considered to be prevalent among army troops and was reported long back in 1934 (4). The disease remains mostly undiagnosed due to lack of awareness among affected persons and non-availability of diagnostic facilities. The major symptoms of scrub typhus are non-specific ranging from high-grade fever of 1–2 weeks, a typical eschar/papule at the site of bite by mite, headache, myalgia, dry cough, lymphadenopathy, hepatosplenomegaly, apathy, breathlessness, and myocarditis. Mortality may reach up to 30% if not treated. The diagnosis is made by Weil–Felix test, Indirect Immunofluorescence Test, Quantitative ELISA, and polymerase chain reaction (15). Scrub typhus is a zoonotic disease and infection of rickettsia is maintained in small rodents in recently cleared forest/scrub, grassy areas. The nymph and adult mite do not feed on man and the transmission to man occurs through larval stage of mite accidentally. Transovarian transmission through larvae has been reported. When the ecological conditions in forest are disturbed due to deforestation, and human beings intrude into cleared forest, the larval mite attack man.

**Figure 1:**
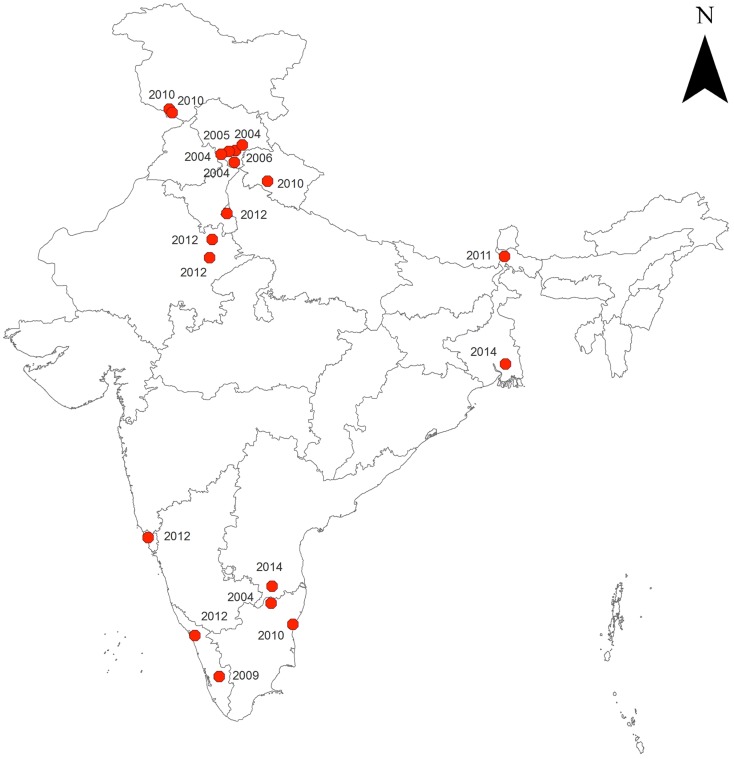
**Reported occurrence of scrub typhus in India (2004 onward)**.

Tsai and Yeh (16) studied the association of climatic/environmental factors with distribution of scrub typhus in Taiwan. They found that some areas exhibit no climatic effect, whereas in another area, the incidence correlates positively with higher temperatures during the warm season, and the third area correlates positively with higher surface temperatures and longer hours of sunshine. The results also showed that scrub typhus is associated with farm worker population density, timber management, recreational forest, natural reserve, or other purpose. Though whole India has been reported to be endemic for scrub typhus (17), however, the reporting of scrub typhus has seen resurgence since 2004 from different parts of India, e.g., Himachal Pradesh (18–22), Darjeeling (23), Jammu and Kashmir (24, 25), Rajasthan (26), Andhra Pradesh (27, 28), Uttarakhand (29), Pondicherry (30), Goa (31), Delhi (32), Kerala (33, 34), and West Bengal (35) (Figure 1).

Figure 1 reveals that the areas reporting scrub typhus are forested or scrubby with moderate to high humidity. Based on the retrospective data, the preferred habitats of rodent reservoirs and mites may be mapped for effective preparedness to prevent the disease.

## Arboviral Diseases

### Japanese encephalitis

Japanese encephalitis is a viral disease transmitted mainly by *Culex triteaniorhynchus*, the *Culex vishnui* group of mosquitoes. The epidemiology involves pig as amplifier host while man is a dead end host^2^. Cattle egrets are also known to act as reservoir of infection. As the vector species prefers to breed in rice fields, the distribution of JE coincides with rice growing areas of eastern Uttar Pradesh, Andhra Pradesh, Tamil Nadu, Brahmaputra Valley in Assam, Karnataka, and West Bengal, etc. In India, the endemicity of JE was confined to seven states in 2008, while in 2011, it spread to 15 states and in 2013, 13 states reported JE cases with 1086 cases^1^ indicating geographical spread. Though the typical biotope of JE endemic area is well defined, the occurrence of acute encephalitis syndrome is complicating the problem owing to ill-defined etiology and risk factors. In 2011, recording of nine cases of JE from Delhi^1^ warrants detailed investigation of ecological features supporting biotope for JE transmission.

### West Nile virus

India is endemic for WNV with reported infection in human beings mainly from southern India. In nature, the natural cycle is maintained in bird and mosquito. Antibodies against WNV were first detected from Bombay in 1952 (5). Pigs and horse have also been found with antibodies against WNV. The symptoms are mild influenza, fever, general body ache, headache, nausea, and vomiting including encephalitis syndrome. The disease is transmitted mainly by *Culex vishnui* group of mosquitoes, and transovarial transmission has been reported. Till 2002, WNV was reported mainly from southern India, but in 2002, Thakare et al. (36) reported prevalence of WNV from non-endemic (Maharashtra and Rajasthan) and endemic area of JE (Goa and Orissa). Khan et al. (37) reported the WNV from Dibrugarh, Golaghat, Sivasagar, and Tinsukia districts of Assam. WNV may be a cause of acute encephalitis syndrome in JE endemic areas. Further studies are needed to delineate the WNV endemic and potential areas for better prevention, particularly in JE non-endemic areas.

### Chikungunya

Chikungunya fever is caused by CHIK virus and transmitted by *Aedes aegypti* mosquito. The disease resembling the symptoms of chikungunya was reported from India in 1824^3^ but the outbreaks due to chikungunya were witnessed in 1963. Transmission cycle of CHIKV can be man–mosquito–man (urban cycle) or animal–mosquito–man (sylvatic cycle). In Africa, the transmission of chikungunya is maintained in a sylvatic cycle, i.e., monkeys, mosquito, and man, while in India man-to-man transmission through *A. aegypti* mosquito is known. *A. albopictus* has also been reported as vector of chikungunya in Africa (38). In Malaysia (39), neutralizing antibodies were reported in wild monkeys in 1960s, providing a clue that CHIK virus may be maintained in nature through animals.

The outbreaks of chikungunya in India were recorded in 1960s and 1970s (40). Banerjee (41) in 1965 revealed that antibodies to CHIK virus were detected in the sera collected in 1956 from Madras state. Resurgence of chikungunya in India was witnessed in 2005 when severe outbreaks were reported from whole southern India and the spatial distribution gradually extended to northern India. Before the outbreaks of 2005, virus activity was detected in 2001 (42) by isolating CHIK virus from *A. aegypti* from Yawat town of Pune district. Since the outbreak in 2005, chikungunya cases have been reported from 13 to 18 states of India with 18,639 clinically suspected cases in 2013^1^. Most of the publications have reported the epidemiological profile of cases, but did not elucidate the reasons of outbreaks from the ecological, climatic, and socioeconomic development points of view. In view of reported role of *A. albopictus* mosquito in transmission, which prefers peri urban areas, there are possibilities of sylvatic cycle of chikungunya in India warranting in depth investigations.

### Crimean–Congo hemorrhagic fever

Crimean–Congo hemorrhagic fever is a viral disease transmitted by ticks of *Hyalomma anatolicum anatolicum* species or through direct contact with reservoir animals or human beings. Persons residing in rural area are at the risk of contracting CCHF as they live in vicinity of livestock and other wild animals like hare and hedgehog, which are reservoirs of the infection. Nosocomial outbreaks among healthcare staff have also been reported. The disease was first of all reported from Crimea, Russia in 1944 and is distributed in Africa, Asia, Europe, and Middle East, and the symptoms are similar to dengue hemorrhagic fever with mortality rate up to 80% during outbreaks (43). The first case of CCHF was reported from Ahmedabad, India in 2010 (6), and in 2014, an outbreak was reported in a cluster in a village in Amreli and Patan district of Gujarat (7). IgG antibody positivity in the animals was up to 43.9% and Hyalomma ticks were also found positive by RT-PCR. There is need to create awareness among public health stakeholders for symptoms, diagnosis, and preventive measures for timely diagnosis and management of CCHF cases and for containing spread.

## Conclusion

Zoonoses are a major challenge to public health in view of rapid deforestation, urbanization, population movement, changing climatic scenarios, etc. Various developed and developing countries have faced unknown as well as re-emerged diseases like chikungunya, scrub typhus, Swine flu, dengue, CCHF and CL. Studies undertaken so far have been of reactive type, offering little solution for preventive aspects in a long-term perspective. In view of life threatening nature of most of the diseases, there is need for understanding having eco-epidemiological approach in respect of each disease with emphasis on habitats of reservoirs of infection, micro niche of arthropod/insect vectors, and vulnerable areas from the climatic determinants point of view for development of pathogen and vectors. As there is no routine surveillance for most of the zoonotic diseases discussed here, emphasis should also be given to periodic serological surveys for detection of evidence of arbovirus infections in vulnerable areas in a systematic way. Reporting of epidemiological data in public domain should also be augmented for neglected diseases like scrub typhus, WNV, CCHF, and CL. With the advent of tools like satellite remote sensing, geographic information system, and mathematical modeling in better understanding of diseases epidemiology, it is possible to detect and identify ecological niche at finer resolutions and map and project the current as well as potential risk of the diseases, which have ecology-driven epidemiology. The risk factors also need to be ascertained for prevention from contracting the diseases. Risk maps of zoonotic diseases discussed here, creation of facilities for laboratory diagnosis of pathogens, awareness in the communities about symptoms of diseases, and health education for source reduction of vectors’ breeding wherever possible, should be thrust areas for preventing vector-borne zoonotic diseases. This review should sensitize public health stakeholders to emphasize on understanding the eco-epidemiology of the discussed zoonotic diseases for taking preventive actions in India, as well as in Southeast Asia.

## Conflict of Interest Statement

The author declares that the research was conducted in the absence of any commercial or financial relationships that could be construed as a potential conflict of interest.

## Footnotes

^1^http://www.nvbdcp.gov.in

^2^http://www.icmr.nic.in/pinstitute/niv/JAPANESE%20ENCEPHALITIS.pdf

^3^http://www.searo.who.int/entity/emerging_diseases/topics/Chikungunya/en
